# Taking the Lead: A Case Report of a Leiomyoma Causing Duodeno-Duodenal Intussusception and Review of Literature

**DOI:** 10.1089/crpc.2016.0001

**Published:** 2016-04-01

**Authors:** Louis F. Chai, Philip M. Batista, Harish Lavu

**Affiliations:** ^1^ Rutgers-Robert Wood Johnson Medical School, Piscataway, New Jersey.; ^2^ Department of Surgery, Jefferson Pancreas, Biliary, and Related Cancer Center, Thomas Jefferson University Hospital, Philadelphia, Pennsylvania.

**Keywords:** leiomyoma, duodeno-duodenal intussusception, gastrointestinal bleed

## Abstract

***Background:*** Duodenal masses are rare entities and symptomatic presentation generally is due to abdominal pain or the presence of gastrointestinal bleeding. A number of published case reports in the literature have detailed various neoplasms that have caused an intussusception isolated to the duodenum. This is a particularly unusual phenomenon due to the location and fixation of this portion of the proximal small bowel to the retroperitoneum. We present here a case of duodeno-duodenal intussusception secondary to a leiomyoma.

***Case:*** A 65-year-old Caucasian male presented with intermittent bloody stools and syncope over a 9-month period secondary to a duodenal leiomyoma causing intussusception, which was treated through a pancreaticoduodenectomy.

***Conclusion:*** Intussusception of the duodenum is an uncommon entity and the diagnosis of a leiomyoma should be considered in the setting of a potential mass in the small intestine.

## Introduction

Etiologies for gastrointestinal (GI) bleeding commonly include bleeding ulcers, arterial venous malformations, diverticular disease, hemorrhoids, and anal fissures, whereas uncommon causes include Meckel's diverticulum and intussusception. Typically, conventional diagnostic modalities such as physical examination, endoscopy, and radiological imaging yield a cause, although occasionally surgical intervention is required to obtain a diagnosis. Here, we report the first case of a patient with GI bleeding secondary to duodeno-duodenal intussusception caused by a leiomyoma.

## Case

A 60-year-old male presented to the Thomas Jefferson University Hospital in August 2015 with a syncopal episode, fatigue, abdominal pain, and dyspnea on exertion. A review of the patient's records revealed two previous hospitalizations to an outside institution over the prior 9 months for melena and near syncope. A computed tomography (CT) with enterography performed at the outside hospital in November 2014 revealed a 4 cm intraluminal mass-like density. An esophagogastroduodenoscopy (EGD) with endoscopic ultrasound was performed at that time, which revealed an extrinsic thickened fold of the second portion of the duodenum (D2), concerning for a duodenal duplication cyst. Fine-needle aspiration of the suspected mass revealed normal villous morphology. At the patient's second presentation to the outside hospital in July 2015, a second EGD evaluation revealed a bleeding ulcer in the stomach and a repeat CT showed that the duodenal mass had increased to 5 cm.

**FIG. 1.**
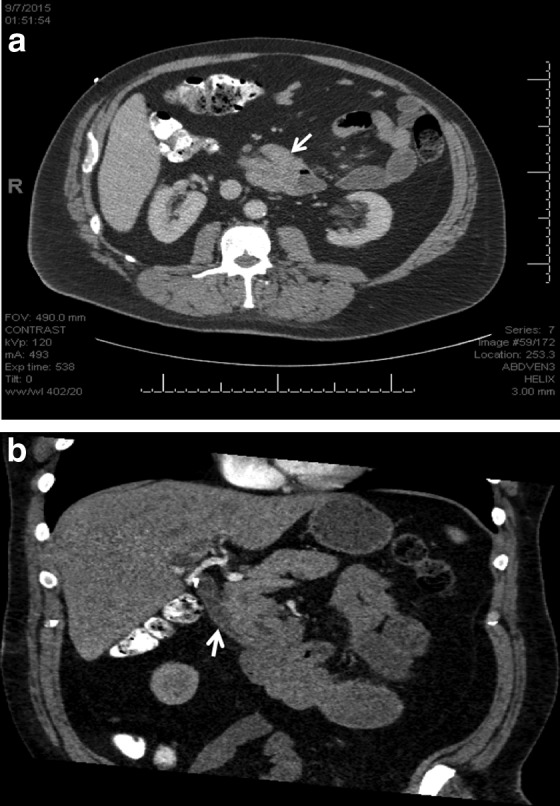
**(a, b)** Axial and coronal CT scan slices showing invagination of proximal portion of duodenum into distal (white arrows). CT, computed tomography.

At the time of transfer to our institution, the patient reported a recent episode of melena without gross blood. He was found to have a hemoglobin of 6.8 g/dL and received two units of packed red blood cells. A CT scan to evaluate the abdominal mass revealed focal dilation and thickening of the third portion of the duodenum (D3) with intussusception of D2 into D3 (Fig. 1). There was also mild intrahepatic biliary dilatation and common bile duct dilatation to 1.2 cm, likely resulting from intussusception of the ampulla of Vater and the distal common bile duct. EGD was performed again, revealing a submucosal lesion along the lateral aspect of D2, causing 80% narrowing of the lumen and erythematous mucosa overlying the lesion. Colonoscopy showed nonbleeding diverticula and a nonbleeding, benign sessile polyp. Capsule endoscopy was nonrevealing.

**FIG. 2.**
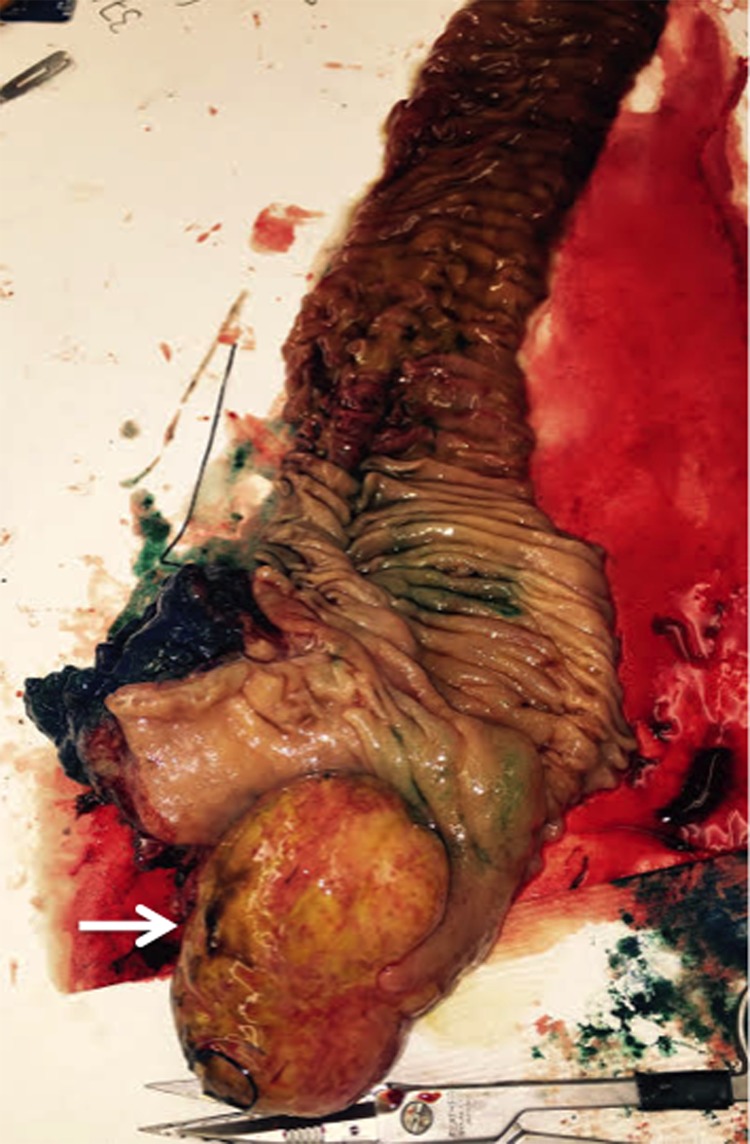
Gross pathology specimen with dissection of duodenum revealing a submucosal leiomyoma (white arrow) measuring 7 × 6 × 3.5 cm.

**FIG. 3.**
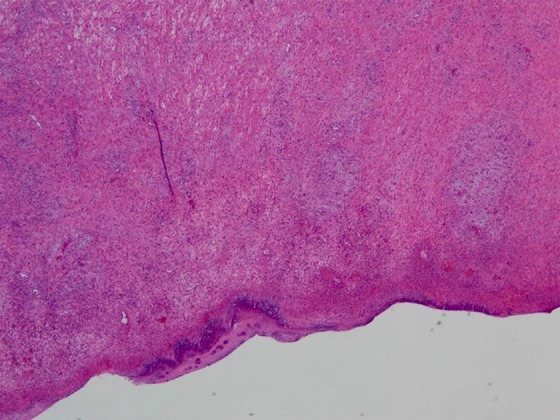
H&E stain of pathological specimen. Analysis revealed mitotic figures less than 5 per 50 high-powered field. Specimen stained positive for actin and desmin, negative for DOG-1, S100, CD34, and ALK-1. H&E, hematoxylin and eosin.

Due to the concern for a malignant mass involving the ampullary complex, a pylorus-preserving pancreaticoduodenectomy was performed. The specimen was removed and gross pathology revealed a 7 × 6 × 3.5-cm duodenal submucosal leiomyoma (Fig. 2). The mass stained positive for actin and desmin and negative for DOG-1, S100, SD34, and ALK-1 (Fig. 3). Negative margins were obtained and none of the 10 harvested lymph nodes were involved with disease. The patient recovered well from surgery, with no further episodes of melena or syncope.

## Discussion

Intussusception is a condition that is more common in children and rarely seen in the adult population. The condition results from the full-thickness invagination of a proximal portion of the bowel into the distal portion.^1^ Presenting symptoms are classically described with the triad of abdominal pain, a palpable abdominal mass, and bloody stools, but all three are clinically found in less than 10% of the population with intussusception.^2^ More commonly, patients present with nonspecific symptoms including intermittent abdominal pain, nausea, vomiting, and anemia.^3^ Given the wide range of diseases with this constellation of symptoms, physicians must have a strong clinical suspicion to make the diagnosis. Work-up may include hemoglobin levels, fecal occult blood tests, cross-sectional imaging (CT or MRI), and endoscopy (EGD, colonoscopy, capsule endoscopy, or push endoscopy). Definitive diagnosis may require operative intervention with surgical exploration of the abdomen.

The most common location for intussusception is the ileocolic junction, followed by ileoileal and colocolic.^4^ Intussusception of the proximal small bowel, especially with the intussusceptum and intussuscipiens isolated to the duodenum, is a rare condition with only 16 reported cases in the literature occurring in 19 total patients (Table 1).^1,2,4–17^ Typically, the anatomic position of the duodenum in the retroperitoneal space and its fixed attachments in this region serve to prevent it from being susceptible to intussusception. However, this condition can occur due to a lead point such as a mass (90% of adults cases)^18^ or secondary to congenital malrotation of the bowel, where the duodenum is more mobile, allowing the intussuception to occur.^4^ The most common etiologies for such lead points include both benign and malignant tumors, lipomas, Brunner's gland hamartomatous polyps, or adenomas.^5^ Although leiomyomas have been known to cause intussusception in other parts of the intestine, our patient represents the first reported case to our knowledge of duodeno-duodenal intussusception secondary to leiomyoma.

**Table 1.** **English Literature Review Highlighted 16 Reports of 19 Total Patients with Confirmed Duodeno-Duodenal Intussusception**

*Study*	*Patient age (years)*	*Presenting symptom(s)*	*Lead point causing intussusception*
Vinnicombe and Grundy^7^	52	Abdominal pain, jaundice	Villous adenoma
O'Connor et al.^8^	32	Abdominal pain, anorexia, vomiting	Duplication cyst
Mishra et al.^4^	18	Abdominal pain and distention, fever, vomiting	None
Gupta et al.^2^	—	—	Adenoma
	—	—	Adenoma
Limi et al.^9^	40	Abdominal discomfort, melena, diarrhea	Brunner's gland adenoma
Abeysekera et al.^10^	67	Abdominal pain, melena	Brunner's gland adenoma
Singla et al.^11^	43	Abdominal discomfort, fatigue, generalized weakness	Brunner's gland adenoma
Blanchet et al.^12^	69	Abdominal pain, nausea, vomiting	Lipoma
Ko et al.^13^	43	Abdominal pain, melena	Duplication cyst
Watanabe et al.^6^	31	Abdominal pain	Tubulovillous adenoma
Naik et al.^1^	35	Abdominal pain, vomiting	Tubulovillous adenoma
Larsen et al.^14^	19	Abdominal pain, nausea, vomiting	Duodenal membrane
Sinhal et al.^15^	50	Abdominal pain and distention, vomiting	Tubulovillous adenoma
Shakhnovich et al.^16^	14	Vomiting	Duplication cyst
Gardner-Thorpe et al.^5^	66	Abdominal pain, lethargy, pruritus	Villous adenoma
Pradhan et al.^17^	—	—	Adenocarcinoma
	—	—	Villous adenoma
	—	—	Tubulovillous adenoma

—, Indicates nonreported data.

Neoplasms of the small bowel as a whole are exceedingly rare, comprising only 1–5% of all GI neoplasms, with only 1–2% of these being malignant.^19–21^ The more frequently encountered malignant small bowel tumors are adenocarcinomas, lymphomas, and neuroendocrine tumors. Rarely seen are GI stromal tumors, leiomyosarcomas, and leiomyomas. Although leiomyomas are the most common benign lesions of the small intestine, they are a small proportion of total small intestine neoplasms. At one institution over a 115-year span, fewer than nine leiomyomas were diagnosed per year, whereas a second report examining 1091 smooth muscle tumors of the small intestine found that only 1% were truly leiomyomas.^21,22^

Leiomyomas are found most often in the jejunum followed by the ileum and lastly the duodenum. They are characteristically well-defined solitary masses and may appear gray or white. Within the small intestine, they can be found intraluminal, extraluminal, or intramural.^22^ Diagnosis may be difficult despite modern imaging modalities and endoscopy, as was demonstrated in this patient. He suffered from symptoms for nearly 10 months before the diagnosis was confirmed through surgical exploration. In the setting of intermittent GI bleed and abdominal pain with no easily identifiable source, it is important to consider the diagnosis of small bowel intussusception in an adult due to a neoplasm.

## Conclusion

This case report is that of GI bleeding due to a rare duodeno-duodenal intussusception secondary to a small bowel leiomyoma. We emphasize the importance of a keen awareness for this diagnosis and thorough work-up. In this scenario, surgical intervention may prove to be both diagnostic and therapeutic as demonstrated here.

## Abbreviations Used

CT: computed tomography
EGD: esophagogastroduodenoscopy
GI: gastrointestinal
H&E: hematoxylin and eosin
MRI: magnetic resonance imaging
